# Seed Germination Mechanism of *Carex rigescens* Under Variable Temperature Determinded Using Integrated Single-Molecule Long-Read and Illumina Sequence Analysis

**DOI:** 10.3389/fpls.2022.818458

**Published:** 2022-03-03

**Authors:** Hui Li, Ke Teng, Yuesen Yue, Wenjun Teng, Hui Zhang, Haifeng Wen, Juying Wu, Xifeng Fan

**Affiliations:** Beijing Academy of Agriculture and Forestry Sciences, Beijing, China

**Keywords:** *Carex rigescens*, seed germination, physiological changes, PacBio and Illumina sequencing, WGCNA

## Abstract

The turfgrass species *Carex rigescens* has broad development and utilization prospects in landscaping construction. However, seed dormancy and a low germination rate have inhibited its application. Furthermore, the molecular mechanisms of seed germination in *C. rigescens* have not been thoroughly studied. Therefore, in the present study, PacBio full-length transcriptome sequencing combined with Illumina sequencing was employed to elucidate the germination mechanism of *C. rigescens* seeds under variable temperatures. In general, 156,750 full-length non-chimeric sequences, including those for 62,086 high-quality transcripts, were obtained using single-molecule long read sequencing. In total, 40,810 high-quality non-redundant, 1,675 alternative splicing, 28,393 putative coding sequences, and 1,052 long non-coding RNAs were generated. Based on the newly constructed full-length reference transcriptome, 23,147 differentially expressed genes were identified. We screened four hub genes participating in seed germination using weighted gene co-expression network analysis. Combining these results with the physiological observations, the important roles of sucrose and starch metabolic pathways in germination are further discussed. In conclusion, we report the first full-length transcriptome of *C. rigescens*, and investigated the physiological and transcriptional mechanisms of seed germination under variable temperatures. Our results provide valuable information for future transcriptional analyses and gene function studies of *C. rigescens*.

## Introduction

The perennial *Carex rigescens* is an ideal turfgrass found in open forests and used for lawns in urban landscaping in northern China (Ma and Mao, 1990; Li et al., 2001; Xue et al., 2005). This species is highly tolerant to cold, shade, drought, heat, and other abiotic stresses (Zhang et al., 2021) and can adapt to a variety of soil conditions (Zhang et al., 2019). Such traits increase the applicability of this species as lawn grass and help alleviate the current dependence on externally sourced lawn grass species in China. However, seed dormancy and low germination rate inhibit the application of this species. Studies on *C. rigescens* have mainly focused on understanding its morphology, physiology, and proteomics under salt stress (Zhang et al., 2020; Zhang et al., 2021) and pharmaceutical applications (Gao et al., 2009) and common methods of breaking seed dormancy aiming to improve its seed germination rate (Liang et al., 2011; Sun et al., 2011). At present, systematic studies on the physiological and molecular mechanisms of *C. rigescens* seed germination are lacking. Fully understanding its seed germination mechanism will help promote the development and application of *C. rigescens* in lawns. Therefore, it is vital to clarify the physiological and molecular mechanisms of *C. rigescens* seed germination.

A series of key events occurs during seed germination, including mobilization of reserves, energy production, signal transduction, and transcriptional activation (Han and Yang, 2015). After the dry seed absorbs water and expands rapidly, it mobilizes internal storage substances (such as proteins, lipids, and starch) to initiate physiological and metabolic processes (Mangrauthia et al., 2016; Bai et al., 2021). According to previous reports, low temperature inhibited seed germination of *Paeonia ostii* “Feng Dan” by inducing ABA synthesis, inhibiting starch degradation, reducing soluble sugar consumption, and disrupting protein synthesis (Ren et al., 2018). *GBSS* and *SUSY* genes are key regulators of sucrose and starch metabolic pathways during *Castanea henryi* seed germination (Liu et al., 2020). The sucrose transporter sut4 interacts with cyb5 to actively mediate sucrose and glucose signals to inhibit Arabidopsis seed germination (Li et al., 2012). *Podophyllum* seeds germinate completely by regulating protein changes (Dogra et al., 2013). *GhHSP24.7* induces reactive oxygen species, which accelerates endosperm rupture (Ma et al., 2019), whereas the B-box protein *BBX19* induces *ABI5* to inhibit seed germination (Bai et al., 2019). However, despite previous research on seed germination mechanisms, to the best of our knowledge, those of *C. rigescens* have yet to be reported.

With the current advance in sequencing technology, it is possible to clarify seed germination mechanism by analyzing the relationship between gene expression and phenotype. PacBio single molecular real time (SMRT) sequencing can avoid assembly errors and provide complete transcript sequence information for species in case of no or poor reference genomes (Workman et al., 2018). To obtain the full-length transcript information of plants and accurately address biological problems, a method combining PacBio SMRT sequencing with Illumina sequencing has been used in several studies: for example, to determine how alternating temperature breaks the morphophysiological dormancy of *Polygonatum sibiricum* seeds (Liao et al., 2021), response to drought stress during seed germination of Adzilla beans (Zhu et al., 2020), and potential transcriptional variants of genes involved in oil biosynthesis in *Camellia oleifera* seeds (Gong et al., 2020). Similar to that in previous studies, this combined method can be used to study *C. rigescens* seed germination and will provide useful genetic information as well as allow elucidation of germination mechanisms.

Given the lack in such research on *C. rigescens*, the aim of this study was to obtain full-length transcripts of *C. rigescens* and explore hub genes involved in seed germination. In addition, in this study, we attempted to identify key metabolic pathways involved in germination in combination with physiological changes. Our findings may provide valuable information for transcriptional analysis and gene function research of *C. rigescens* in the future.

## Materials and Methods

### Plant Materials and Seed Germination Test

In May 2020, we collected seeds of *C. rigescens* at the National Experiment Station for Precision Agriculture (Beijing, China). Seeds with full grains were selected and soaked in 10% sodium hypochlorite for 30 min for disinfection, after which they were washed with distilled water five or six times. The cleaned seeds were seeded in 12 cm × 12 cm × 5 cm germinating boxes lined with three layers of filter paper. The germinating box was incubated in a growth chamber (RXZ-380D-LED; Ningbo Jiangnan Instrument Factory, Ningbo, China) with an 8/16-h (day/night) photoperiod, average 24,000 Lx, 80% relative humidity, and either 30/20°C (day/night) or 20°C (all day). During germination, the filter paper was kept wet. When the radicle broke through the seed coat and exceeded half the seed length, we began counting the germination number once a day. When the seed germination number remained unchanged for three consecutive days, the germination rate, germination potential, germination index, and mean germination time were determined at the end of the germination test.

### Determination of Nutrient Content and Enzyme Activity

The nutrient content and enzyme activity of five different germination stages of *C. rigescens* seeds were measured at 30/20°C. To determine the related physiological indexes using spectrophotometry, kits for plant soluble sugar, plant starch, and plant soluble protein contents and peroxidase activity (Suzhou Keming Biotechnology Co., LTD., Suzhou, China) were used. To determine amylase activity, 3,5-dinitrosalicylic acid was used (Shi and Yang, 1999). Three biological replicates were used for physiological measurements.

### RNA Sample Preparation

**FIGURE 1:**
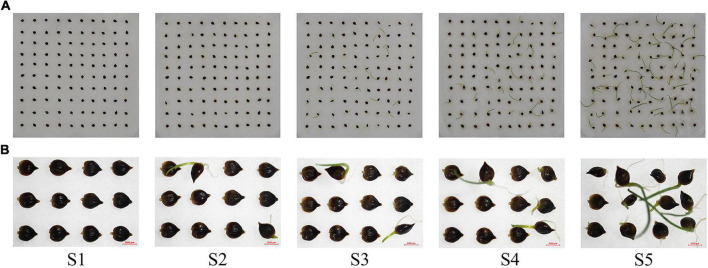
Seed germination status of *Carex rigescens*. **(A)** Seed germination of *C. rigescens* at different germination stages. **(B)** Part germination of *C. rigescens* seeds at different germination stages.

The seed pretreatment and environmental (light and humidity) conditions for germination were consistent with those used for the germination test. The difference in germination rate at different germination stages at 30/20°C was used as the sampling standard. Samples were collected at 3 d (S1-germination rate, 0), 7 d (S2-germination rate, 10%), 9 d (S3-germination rate, 30%), 10 d (S4-germination rate, 50%), and 13 d (S5-germination rate, 80%) after seeding (Figure 1). The seeds from three germinating boxes were randomly selected each time as three replicates, quickly frozen using liquid nitrogen, and stored at −80°C for subsequent experiments. Total RNA was extracted using a plant RNA kit (OMEGA, GA, United States). The quantity and integrity of the RNA samples were assessed using a Drop ND-1000 spectrophotometer (NanoDrop Technologies, Wilmington, DE, United States) and 2100 Bioanalyzer (Agilent Technologies, Santa Clara, CA, United States), respectively.

### PacBio and Illumina cDNA Library Preparation and Sequencing

Libraries were constructed after the qualified samples had been tested. For PacBio cDNA library preparation, the full-length cDNA of total RNA (evenly mixed RNA from S1 to S5) was synthesized using SMARTer™ PCR cDNA Short Kit (TaKaRa, Dalian, China). Then, the full-length cDNA was amplified, repaired, and connected to a dumbbell-shaped SMRT to obtain a sequencing library with a size of 1–6 Kb. The library was generated by Biomarker Technology Co. (Biomarker, Beijing, China). For Illumina cDNA library construction, 15 mRNA samples were first enriched with oligo (dT) magnetic beads and reverse transcribed. Then, the double-stranded cDNA was repaired at the end, a tail was added, and the connector was connected. Finally, the cDNA library was amplified using PCR. QSEP400 (BiOptic Inc., Taiwan, China) was used to assess the quality of the library. The fragment sizes of the PacBio and Illumina cDNA libraries met the requirements of sequencing reads and library characteristics, and there was no heteropeak pollution. In addition, the following criteria were met: the amount of the Illumina cDNA library must be sufficient to opera the machine twice, and the concentration must be more than 3 ng/ml; and PacBio full-length cDNA concentration must be more than 20 ng/ml. Then Illumina Hi-Seq4000 (San Diego, CA, United States) and Pacific Bioscience Sequel (Pacific Biosciences, CA, United States) platforms were used for next generation sequencing and SMRT sequencing, respectively.

### PacBio and Illumina Sequencing Data Analysis

Clean data were obtained by filtering low-quality data from the original dataset and intercepting sequencing connectors and primer sequences in reads. The circular consensus (CCSs) were extracted from the original sequence according to the following conditions: full passes ≥3 and sequence accuracy >0.9. Meanwhile, the CCSs were polished. Among them, sequences with the correct 5′ primers, 3′ primers, and polyA tail were full length and were further divided into chimeric and non-chimeric sequences according to their position relationships. Then, the Iso Seq module in SMRT Link software (Yang et al., 2020) was used to cluster sequences similar to the full-length non-chimeric (FLNC) sequences; each cluster obtained a consensus isoform. Sequences with an accuracy >99% were high-quality transcripts, and the rest were low-quality transcripts. Next, consistently low-quality sequences obtained from each sample were corrected with the corresponding Illumina RNA-seq data using Prooverd software (Hackl et al., 2014) to improve the accuracy of the sequences. The CD-HIT software (Li and Godzik, 2006) was used to merge the sequences with high similarity and remove redundant sequences, and non-redundant transcript sequences were obtained for subsequent analysis.

### qRT-PCR Analysis

The 15 extracted RNA samples were reverse-transcribed into cDNA, and the product was quantified using the SYBR Green PCR Master mix (TaKaRa, Dalian, China) in Bio-Rad CFX Connect Thermal Cycler (Bio-Rad, CA, United States) under the following conditions: 95°C for 10 min, followed by 40 cycles at 95°C for 15 s, 60°C for 1 min, and 65°C for 5 s. Ten differentially expressed genes (DEGs) were randomly selected, primers were designed using Primer Premier 5 software,^1^ and cDNA was diluted with RNase-free water to a concentration of 1:3. The *C. rigescens* SAND gene (Zhang et al., 2019) (SAND family protein, GenBank Accession No.: 817387) was used as the reference. Relative gene expression levels were normalized using the 2^–Δ^ ^Δ^ ^CT^ method and setting the expression of S1 to 1 (Livak and Schmittgen, 2001). The primers used for gene expression analysis are listed in Supplementary Table 1.

### Prediction of Alternative Splicing, Coding, and Long Non-coding RNA Sequences

Alternative splicing (AS) events were predicted for transcripts after deredundancy. All sequences were pairwise compared using the BLAST software (Altschul et al., 1997). In the comparison results, a variable splicing event was considered to have occurred when both sequences were longer than 1,000 bp and there were two high-scoring segment pairs, allowing all variable transcripts to overlap by 5 bp, while the gap in variable splicing was >100 bp and the distance between 3′ and 5′ ends was at least 100 bp (Liu et al., 2017). The identified AS events were analyzed using Kyoto Encyclopedia of Genes and Genomes (KEGG). According to the length of the open reading frame (ORF), log-likelihood score, and the comparison between amino acid sequence and protein domain sequence in the Pfam database, transcoder software can identify reliable coding sequences (CDS) from transcript sequences. Long non-coding RNA (lncRNA) is another significant component of the transcriptome. The coding potential calculator, coding-non-coding index, Pfam protein domain analysis, and coding potential assessment tool were used to screen transcripts for coding potential, and the transcripts with coding potential were filtered out. The intersection of the above non-coding transcripts was obtained for subsequent lncRNA analysis.

### Functional Annotation

Annotation information of transcripts was obtained by comparing the obtained non-redundant transcripts sequences with those in the NR, Swiss-Prot, gene ontology (GO), clusters of orthologous groups (COG), euKaryotic ortholog groups (KOG), protein family (Pfam), and Kyoto Encyclopedia of genes and genomes (KEGG) databases using BLAST software (Altschul et al., 1997) (Version 2.2.26).

### Weighted Gene Co-expression Network Analysis

RNA-Seq by expectation maximization (RSEM) software (Li and Dewey, 2011) was used to quantify the expression level of the transcript through the mapped read location information on the third-generation transcript. The FPKM method was used to normalize the number of mapped reads and transcript length in the sample to measure the transcript or gene expression level. Using fold change ≥2 and false discovery rate <0.01 as criteria, DESeq2 (Robinson et al., 2010) was used to screen DEGs during *C. rigescens* seed germination. Owing to the large number of samples in this study (n = 15), we used the weighted gene co-expression network analysis (WGCNA) online tool of the BMK Cloud Platform^2^ to analyze the DEGs. An undirected network for the FPKM of the DEGs was constructed using the soft threshold method to obtain an appropriate soft threshold. The dynamic programming algorithm was used to divide the clustered genes into modules, with 30 as the minimum module size, and to merge the modules, with 0.25 as the minimum height of the merged modules, to obtain the final module. The gene expression patterns of the obtained modules were analyzed, and GO and KEGG enrichment analyses were performed.

Hub gene screening was completed using the Cytoscape software.^3^ The kME value can be used to evaluate effective connectivity between genes. In this study, the kME of genes in each module was calculated, and genes with kME > 0.7 were regarded as module members to represent the expression trend of the module. Then, the top 150 genes with high kME values in each module were correlated to obtain the weight value of the correlation between two genes. The top 500 gene combinations with higher weight values were finally selected to construct a visual interaction network in Cytoscape.

### Statistical Analysis

Germination and physiological index data were statistically analyzed using independent-samples *T*-test (two-tailed, *P* < 0.01) and one-way ANOVA (*P* < 0.05) in IBM SPSS Statistics 23 (IBM, Chicago, IL, United States), respectively. All analyses had three biological replicates. Data visualization was performed using the Origin Pro V. 2019B software (OriginLab Corporation, Northampton, MA, United States).

## Results

### Seed Germination Status of *Carex rigescens* Under Variable Temperature

**FIGURE 2:**
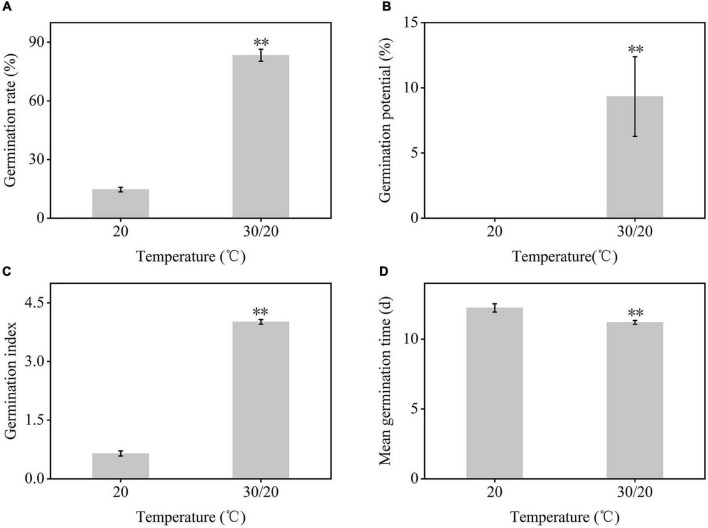
Germination indexes of *Carex rigescens* seeds under different temperature conditions. **(A)** Germination rate. Germination rate (%) = Number of final seed germination/number of tested seeds × 100%. **(B)** Germination potential. Germination potential (%) = Number of seeds germinated in the first 3 days of the germination/number of tested seeds × 100%. **(C)** Germination index. Germination index = Σ (Gt/Dt), where Gt is the number of germinated seeds in a day, and Dt is the corresponding germination days. **(D)** Mean germination time. Mean germination time (d) = Σ nt/Σ n, where *n* is the number of newly germinated seeds on day t, and t is the number of germination days. Data are expressed as mean ± SD (*n* = 3). ^**^Indicates that the germination indexes between variable temperature and constant temperature were significantly different at *P* < 0.01 based on independent-samples *T*-test.

The germination of *C. rigescens* seeds responded differently to different temperature conditions (Figures 1, 2). The germination rate and germination index of *C. rigescens* under variable temperature conditions were significantly higher than those at constant temperature (*P* = 0.0000034 and *P* = 0.0000003, respectively). Under variable temperatures, the germination rate was 5.68 times higher than that at constant temperature. Furthermore, the germination potential was significantly different between the two temperature conditions (*P* = 0.0061223). The mean germination time of seeds under variable temperature was shortened by 1.04 days.

### Dynamic Changes in Nutrient Contents and Enzyme Activities During Seed Germination

**FIGURE 3:**
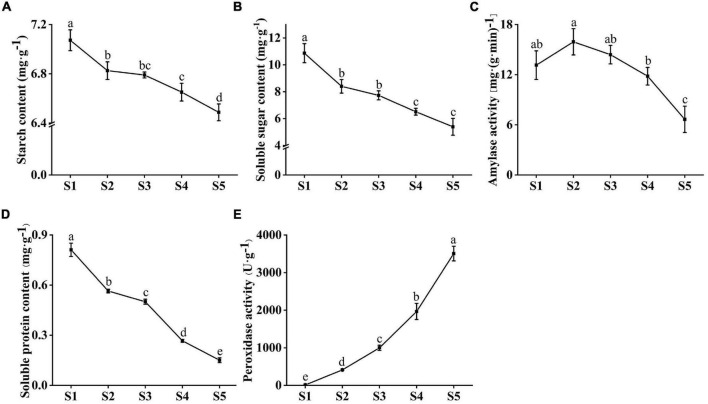
Changes in physiological indexes of *Carex rigescens* seeds at five different germination stages under variable temperature. **(A)** Starch content. **(B)** Soluble sugar content. **(C)** Amylase activity. **(D)** Soluble protein content. **(E)** Peroxidase activity. Different small letters indicate that physiological indexes at different seed germination stages significantly different (*P* < 0.05) based on one-way ANOVA.

The starch and soluble sugar content of *C. rigescens* seeds decreased during germination (Figures 3A,B). The starch content in any two periods was significantly different except for S2 vs S3 and S3 vs S4. The soluble sugar content was significantly different between stages except for S2 vs S3 and S4 vs S5. Amylase activity plays an important role in the metabolism of starch and sugar. With seed germination, amylase activity fluctuated in the range of 6.65–15.93 mg⋅(g min)^–1^ and reached the highest value of 15.93 mg⋅(g min)^–1^ in S2 (Figure 3C). There was no significant difference in amylase activity among S1–S3, and the amylase activity in S5 was significantly lower than that in the other stages. During seed germination, soluble protein content decreased from 0.81 mg g^–1^ to 0.15 mg g^–1^, and the content of any two stages reached a significant level (*P* < 0.05, Figure 3D). In addition, we investigated the changes in peroxidase activity related to the pentose phosphate pathway and antioxidant capacity during seed germination (Figure 3E). With the increase in germination number, pod activity increased from 12.59 U g^–1^ to 3506.67 U g^–1^.

### Verification of Gene Expression by qRT-PCR

To verify the reliability of the transcriptome data, 10 genes were randomly selected for real time quantitative RT-PCR analysis (Supplementary Table 2). Among them, four genes were related to photosynthesis, and the other six were involved in different metabolic pathways. Pearson correlation analysis showed that qRT-PCR was consistent with the RNA-seq results, which verified the accuracy and reliability of the RNA-seq data.

### PacBio Sequencing Reads

To obtain full-length reference transcripts from *C. rigescens* with high confidence, we selected seeds at five different germination stages under variable temperature conditions as mixed samples for PacBio SMRT sequencing. The cDNA library size was 1–6 kb. According to the condition of full passes ≥3 and sequence accuracy >0.9, 173,955 CCS reads with mean read length of 2,349 bp were extracted from the raw data (Table 1 and Supplementary Figure 1A), The distribution of full passes of CCS is shown in Supplementary Figure 1B. CCS sequences were divided into full-length and non-full-length sequences depending on whether they contained the correct 5′ primer, 3′ primer, and poly A tail. By detecting the position relationship of the inserted sequences, we obtained 156,750 FLNCs with a mean read length of 2,185 bp, accounting for 90.11% of the total CCS sequences (Table 1 and Supplementary Figure 1C). Through the IsoSeq module in SMRTLink, similar sequences in the FLNC sequence were clustered, and each cluster represented a consensus isoform. In total, 62,094 consensus isoforms were obtained with a mean read length of 2,182 bp, including 62,086 high-quality isoforms and eight low-quality isoforms (Table 1). The length of the consensus isoform varied with the length of the

**TABLE 1:** Single molecular real time sequencing statistics.

Samples	Mix
cDNA size	1-6K
CCS number	173,955
Read bases of CCS	408,628,637
Mean read length of CCS	2,349
Mean number of passes	56
Number of full-length non-chimeric reads	156,750
Mean read length of consensus isoforms	2,185
Number of consensus isoforms	62,094
Average consensus isoforms read length	2,182
Number of polished high-quality isoforms	62,086
Number of polished low-quality isoforms	8

cDNA (Supplementary Figure 1D). The PacBio sequencing reads were longer and the sequence accuracy was lower than that of Illumina sequencing short reads, so we corrected the low-quality sequences obtained from each sample based on Illumina sequencing data of 15 samples with three repetitions in five germination stages. The sequences with high similarity were combined using CD-HIT to remove redundant sequences in the transcripts. Finally, 40,810 non-redundant transcripts of *C. rigescens* were obtained.

### Coding Sequence, Long Non-coding RNA and Alternative Splicing Analysis

**FIGURE 4:**
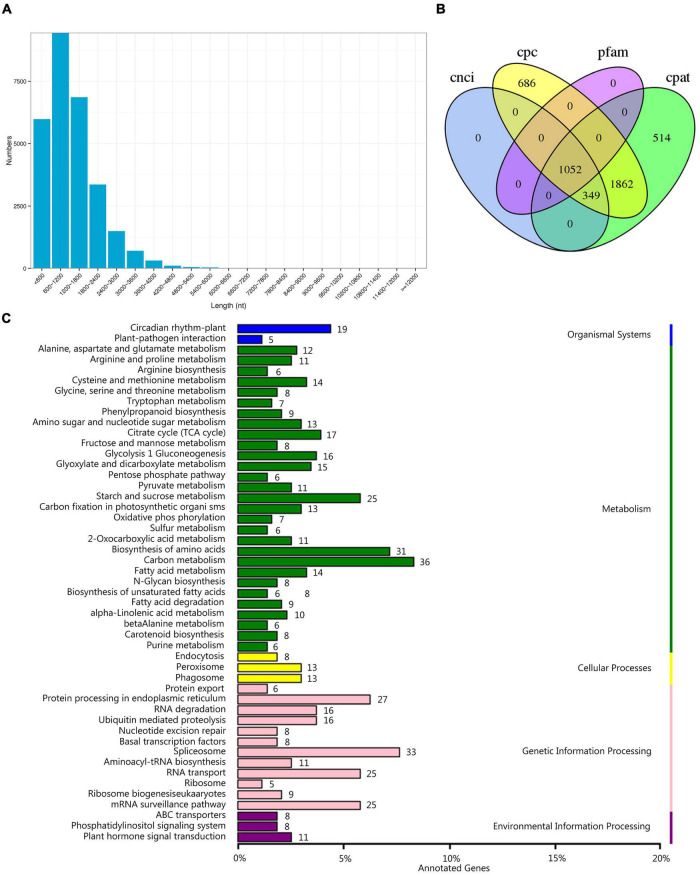
Prediction of coding sequences (CDSs), long non-coding RNAs (lncRNAs) and alternative splicing (AS). **(A)** Distribution of CDSs with a complete open reading frame. **(B)** Venn diagram of the number of lncRNAs predicted. Cpc: coding potential calculator, cnci: coding-non-coding index, Pfam: Pfam protein domain, cpat: coding potential assessment. **(C)** Kyoto Encyclopedia of genes and genomes pathways of genes related to AS. Value to the right of the bar indicates the number of transcripts involved in the metabolic pathway.

The TransDecoder program was used to identify reliable potential CDSs from transcription sequences based on ORF length, log-likelihood score, amino acid sequence, and protein domain sequence alignment. In total, 40,079 ORFs were obtained, including 28,393 complete ORFs. The length distribution showed that 9,447 transcripts with a length of 600–1,200 bp were the most abundant (Figure 4A), accounting for 33.27% of the identified CDSs. Next, the coding potential calculator, coding-non-coding index, Pfam protein domain analysis, and coding potential assessment tool were used to screen the coding potential of transcripts, and 1,052 long non-coding RNAs (lncRNAs) were predicted (Figure 4B). LncRNA and mRNA act through base complementation and pairing. By using the LncTar target gene prediction tool, 1,049 lncRNAs were successfully predicted (Supplementary Table 3). We detected 1,675 AS events. The type of AS could not be determined because there is no reference genome for *C. rigescens*. However, since AS is an important mechanism for regulating gene expression and producing proteome diversity, we showed the results of these AS events in the KEGG enrichment analysis and found that genes involved in carbon metabolism (36), spliceosome (33), biosynthesis of amino acids (31), protein processing in endoplasmic reticulum (27), and starch and sucrose metabolism (25) were highly enriched (Figure 4C).

### Functional Annotation of the Full-Length Transcriptome

**FIGURE 5:**
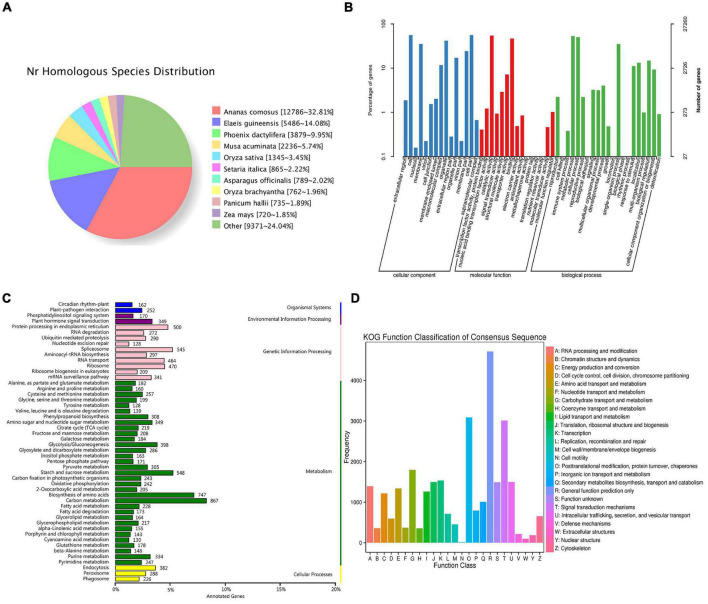
Functional annotations for *Carex rigescens* transcripts. **(A)** NR homologous species distribution diagram of transcripts. **(B)** Distribution of gene ontology terms for all annotated transcripts. **(C)** Kyoto encyclopedia of genes and genomes pathway classification of transcripts. **(D)** EuKaryotic ortholog groups function classification of transcripts.

To comprehensively annotate the transcriptome of *C. rigescens*, 40,810 non-redundant transcripts were functionally annotated by searching COG, GO, KEGG, KOG, Pfam, SwissProt, eggNOG, and NR databases. Among them, 39,315 (96.34%) transcripts were successfully matched with at least one of the eight databases, with a success rate of 45.56–96.34%. Each database annotated 18,519, 27,260, 19,024, 26,177, 34,253, 29,139, 38,687, and 39,016 transcripts, respectively (Table 2). To determine species homologous to *C. rigescens*, the sequences were compared with those in the NR database. The highest homology was observed with *Ananas comosus* (32.81%), followed by *Elaeis guineensis* (14.08%), *Phoenix dactylifera* (9.95%), and *Musa acuminata* (9.95%), while the remaining 37.42% of the sequences were in other plants (Figure 5A).

**TABLE 2:** Annotated transcripts numbers based on eight databases.

Annotated database	Isoform number	300 < = Length < 1,000	Length > = 1,000	Percentage (%)
COG_Annotation	18,591	892	17,695	45.56
GO_Annotation	27,260	1,849	25,399	66.80
KEGG_Annotation	19,024	1,244	17,767	46.62
KOG_Annotation	26,177	1,441	24,725	64.14
Pfam_Annotation	34,253	1,961	32,288	83.93
Swissprot_Annotation	29,139	1,737	27,384	71.40
eggNOG_Annotation	38,687	2,466	36,195	94.80
nr_Annotation	39,016	2,517	36,472	95.60
All_Annotated	39,315	2,559	36,725	96.34

The functional properties of the genes and gene products of *C. rigescens* were comprehensively described according to the GO database. In total, 27,260 transcripts were divided into three functional categories: cellular components, molecular functions, and biological processes, of which 22,205 genes had two or more functions (Supplementary Table 4). The cellular components category was further divided into 15 subcategories, of which the most representative were “cell” and “cell part.” The molecular functions category was also divided into 15 subcategories, with the largest being “catalytic activity” followed by “binding.” “Metabolic process,” “cellular process,” and “single organism process” were the most important of the 21 biological processes (Figure 5B).

According to KEGG annotation, 19,024 transcripts were classified, and “environmental adaptation,” “signal transduction,” “translation,” “carbohydrate metabolism,” “transport and catabolism,” “endocrine,” and “metabolic diseases” were the most abundant subcategories among the six categories of “organismal systems,” “environmental information processing,” “genetic information processing,” “metabolism,” “cellular processes,” and “human diseases” (Supplementary Table 5). The pathway with the greatest number of transcripts was “carbon metabolism” (ko01200), followed by “biosynthesis of amino acids” (ko01230) and “starch and sucrose metabolism” (ko00500) (Figure 5C and Supplementary Table 5).

KOG annotation showed that 26,177 transcripts were assigned to 25 functional clusters, with the largest category being “General functional prediction only” (4,711), followed by “Post-translational modification, protein turnover, Chaperones” (3,087) and “Signal transduction mechanisms” (3,010) (Figure 5D and Supplementary Table 6).

### Weighted Gene Co-expression Network Analysis of Differentially Expressed Genes During Seed Germination

**FIGURE 6:**
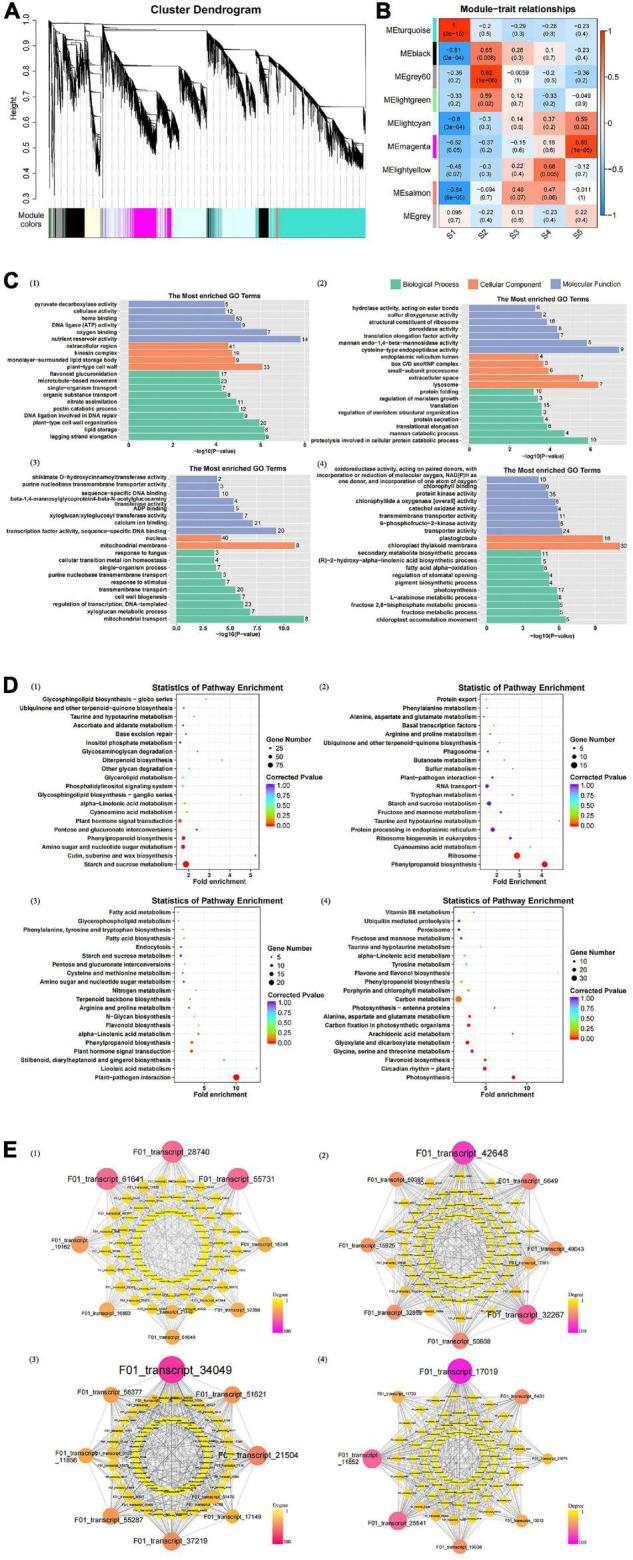
Weighted gene co-expression network analysis (WGCNA) model of differentially expressed genes. **(A)** Hierarchical cluster tree showing co-expression modules identified by WGCNA. **(B)** Module–germination stage relationships. The upper value in the box indicates the correlation coefficient between the module and the germination stage, and the lower value indicates the significance between the two. **(C)** The most enriched gene ontology terms in target modules. The figure in each bar represents the number of genes enriched on the GO term. **(D)** Statistics of significant enrichment of pathways in target modules. **(E)** Potential regulatory network of DEGs in target module. Degree indicates the number of lines owned by a node. A line connects two different genes. Note: (1–4) show the turquoise, gray60, lightyellow, and magenta modules, respectively.

With fold change ≥ 2 and false discovery rate <0.01 as screening conditions, 23,145 DEGs were identified. The seed germination rate was 0 in S1 but increased gradually in S2–S4. The differentially expressed transcripts between the two samples showed that S1vsS5 had the most DEGs, of which 10,883 were upregulated and 6,813 were downregulated. Comparing seeds between the germination and non-germination stages, the number of genes in the differentially expressed transcript set was in the following order: S1vsS5 > S1vsS4 > S1vsS3 > S1vsS2, which was consistent with the phenotypic results (Supplementary Table 7). After seed germination, the number of DEGs in any two stages significantly decreased, indicating that the gene expression levels involved in seed germination may decrease during seed germination. Solving the seed germination mechanism of *C. rigescens* by pairwise comparison is complicated. In the present study, DEGs from 15 samples during seed germination were analyzed by WGCNA to further identify the genetic components involved in seed germination under variable temperature. The results showed that these genes were clustered into nine modules (Figure 6A). The turquoise module was significantly positively correlated with S1 (*r* = 1, *P* = 2e-15), the gray60 module was significantly positively correlated with S2 (*r* = 0.92, *P* = 1e-06), the light-yellow module was significantly positively correlated with S4 (*r* = 0.68, *P* = 0.005), and the magenta module was significantly positively correlated with S5 (*r* = 0.89, *P* = 1e-05) (Figure 6B).

The GO analysis of gene modules showed that four modules highly correlated with S1, S2, S4, and S5 produced significantly enriched GO terms (Supplementary Table 8). In the turquoise module (Figure 6C), the terms related to cell wall were significantly enriched, such as “plant-type cell wall organization,” “pectin catabolic process,” “plant-type cell wall,” “cellulase activity,” and “pyruvate decarboxylase activity.” In addition, DNA ligation involved in DNA repair was significantly enriched. The results showed that the seeds began to swell, the structure and hardness of the seed coat may be changed, and the DNA was repaired to prepare for seed germination. GO terms significantly enriched in the gray60 module included “translation,” “structural constituent of ribosome,” “proteolysis involved in cellular protein catabolic process,” and “protein folding,” which indicates that protein metabolism may play an important role in seed germination (Figure 6C). In the light-yellow module, “nucleus,” “regulation of transcription, DNA-templated,” and “calcium binding” were significantly enriched (Figure 6C). GO terms related to photosynthesis, such as “chloroplast thylakoid membrane,” “chlorophyllide a oxygenase [overall] activity,” and “photosynthesis,” were particularly abundant in the magenta module (Figure 6C).

The KEGG enrichment analysis identified metabolic pathways that may be related to seed germination, of which 20 pathways were significantly enriched (Supplementary Table 9). “Plant–pathogen interaction,” “photosynthesis,” “starch and sucrose metabolism,” “circadian rhythm–plant,” “cutin, suberine, and wax biosynthesis,” and “phenylpropanoid biosynthesis” were the first six pathways of significant enrichment. The “starch and sucrose metabolism” in S1 was particularly abundant, followed by “amino sugar and nucleotide sugar metabolism” (Figure 6D, turquoise module). “Phenylpropanoid biosynthesis” was significantly enriched in S1 and S2, and the enrichment effect strengthened with time (Figure 6D, gray60 module). “Linoleic acid metabolism” was the largest enrichment factor in S4, and “plant hormone signal transduction” was significantly enriched during this process (Figure 6D, light yellow module). Consistent with the GO results, KEGG enrichment at S5 indicated that seedling photosynthesis might be enhanced. As shown in the magenta module (Figure 6D), “photosynthesis,” “carbon fixation in photosynthetic organisms,” and “photosynthesis-antenna proteins” were significantly enriched. In addition, “circadian rhythm–plant” was enriched in this process, which indicated that diurnal light changes may provide sufficient conditions for seedling photosynthesis.

In gene interaction networks, we focused on the top eight genes with a high degree of connectivity in each module (Figure 6E and Supplementary Table 10). Among the 32 genes, six may be related to seed germination: F01_transcript_5649 (*mhsp70*, heat shock 70 kDa protein), F01_transcript_17149 (*CML19*, putative calcium binding protein), F01_transcript_51621 (*CML19*), F01_transcript_17019 (*BBX24*, B-box zinc finger protein 24), F01_transcript_25541 (*KNOX3*, homeobox protein knotted-1-like 3), and F01_transcript_11733 (*KNOX3*) (Figure 6E and Supplementary Table 10).

### Dynamic Analysis of Sucrose and Starch Pathways During Seed Germination

**FIGURE 7:**
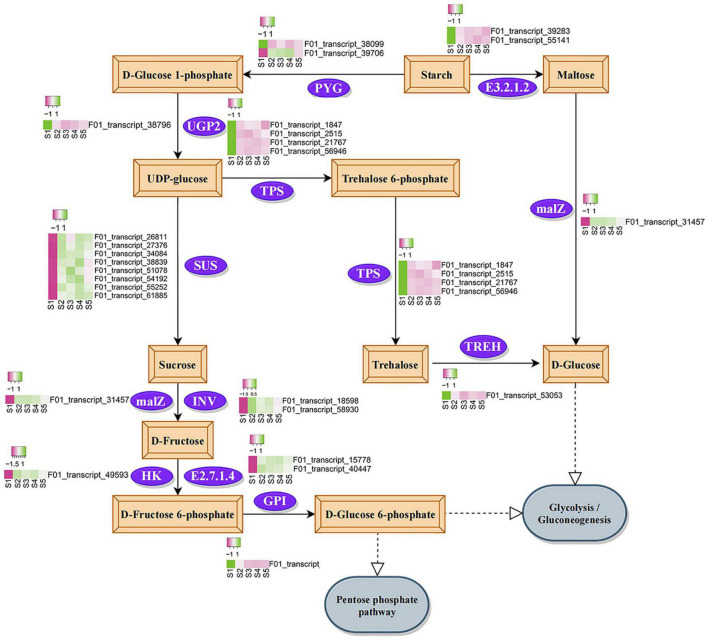
Annotated diagram of differential expression of Kyoto Encyclopedia of genes and genomes sucrose and starch metabolic pathways. The purple box indicates low gene expression, and the green box indicates high gene expression. The meaning of the label is to process the value of each row separately to make it conform to the standard normal distribution. *PYG*, glycogen phosphorylase; *UGP2*, UTP-glucose-1-phosphate uridylyltransferase; *SUS*, sucrose synthase; *malZ*, alpha-glucosidase; *INV*, beta-fructofuranosidase; *HK*, hexokinase; *E2.7.1.4*, fructokinase; *GPI*, glucose-6-phosphate isomerase; *TPS*, trehalose 6-phosphate synthase/phosphatase; *TREH*, alpha, alpha-trehalase; *E3.2.1.2*, beta-amylase.

Combined with the changes in physiological indexes during seed germination, we were interested in the enrichment of sucrose and starch metabolism KEGG pathways in specific modules. During germination, most genes involved in the sucrose and starch metabolic pathways were significantly upregulated (Figure 7). In the process of sucrose synthesis, the expression of one glycogen phosphorylase (*PYG*) gene and eight sucrose synthase (*SUS*) genes increased after seed germination, and the expression of three *SUS* genes was significantly higher than that of other genes (Supplementary Table 11). Sucrose was finally metabolized into D-glucose 6-phosphate under the action of α-glucosidase (*malZ*), beta-fructofuranosidase (*INV*), hexokinase (*HK*), fructokinase (*E2.7.1.4*), and glucose-6-phosphate isomerase (*GPI*). In this process, the gene expression of *malZ*, *INV*, *HK*, and *E2.7.1.4* increased after seed germination, and the expression of *E2.7.1.4* increased the most (Supplementary Table 11). The upregulated expression of *malZ* also regulated the conversion of starch to D-glucose. In addition, UDP-glucose transformed from starch was found to metabolize into trehalose under the downregulated expression of trehalose 6-phosphate synthase/phosphatase (*TPS*), and then converted into D-glucose through the downregulated expression of alpha-trehalase (*TREH*). In conclusion, the expression levels of most enzyme-encoding genes in the sucrose and starch metabolism pathways increased during seed germination.

## Discussion

In the present study, we carried out PacBio single-molecule long-read sequencing to explore the full-length transcriptome of *C. rigescens* and elucidated the underlying regulatory mechanisms of seed germination by analyzing its Illumina short read sequences. After clustering, correction, and redundancy elimination, we finally obtained 40,810 high-quality non-redundant transcripts, which provided the SMRT sequencing map of the full-length transcriptome of *C. rigescens* seeds for the first time.

Long non-coding RNAs, non-protein coding transcripts with a length of more than 200 bp, can affect the expression of flanking genes, change the characteristics of DNA-binding proteins, and participate in a variety of physiological processes (Wilusz et al., 2009). In the present study, 1,052 lncRNAs were predicted based on the SMRT sequencing data. *AtR8* (Li et al., 2015a), *asDOG1* (Fedak et al., 2016), and *BoNR8* (Wu et al., 2019) lncRNAs have been identified as germination-related lncRNA gene. Based on the accuracy and running speed of LncTar for predicting lncRNA targets (Li et al., 2015b), we successfully predicted target genes for 1,049 lncRNAs, which could be candidate for future functional identification.

With its ability to yield multiple proteins from the same gene in eukaryotic genomes, AS serves as an effective mechanism to increase the diversity, plasticity, and elongation of the transcriptome and proteome (Zhang et al., 2016). The expression of variants of snRNP (Raab and Hoth, 2007), *AtPRP4* (Raab and Hoth, 2007), *DOG1* (Bentsink et al., 2006), SnRK2 (Zhang et al., 2016), *At-DRM1* (Tognacca et al., 2019), and *VP1* (Zhang et al., 2016) revealed that AS may play an important role in seed germination. In the present study, 1,675 AS events were detected. Since no genome sequence of *C. rigescens* is available, we were not able to determine the type of AS events, but our KEGG analysis results will provide a reference for further research.

The functional annotation of full-length transcripts, including 39,315 integrated annotated transcripts, provide a reference transcriptome for *C. rigescens*. NR alignment results showed that 32.81% of sequences were highly homologous to those of *Ananas comosus*, indicating that the protein comparison between *C. rigescens* and *A. comosus* was the closest. This is consistent with the findings of our previous study on *Carex breviculmis* (Teng et al., 2019). In the APG IV system, *A. comosus* belongs to Bromeliaceae but *C. rigescens* belongs to Cyperaceae, both belonging to the order Poales. This may be due to the lack of annotation information for many Poales species: *C. rigescens* and *A. comosus* are the closest species. The NR database may also contain insufficient data on species related to Cyperaceae, reflecting the importance of improving the genetic database for this genus.

Weighted gene co-expression network analysis is a powerful tool for identifying modules of highly correlated genes or metabolites based on transcriptome sequencing (Langfelder and Horvath, 2008). In the present study, four germination-related genes, *BBX24*, *KNOX3*, *MHSP70*, and *CML19*, were identified as possible hub genes that regulate seed germination. BBX proteins belong to the zinc finger transcription factor superfamily and play a central role in *Arabidopsis thaliana* seed germination (Strayer et al., 2000); for example *MdBBX10* overexpression improved the germination rate by enhancing reactive oxygen species removal (Liu et al., 2019). According to previous reports, *KNOX4* and *KNAT7* regulate seed coat function during *A. thaliana* seed germination (Bhargava et al., 2013). *KNOX4-like* genes may protect embryos in species without physiological dormancy (Chai et al., 2016). In *Medicago truncatula*, *MtKNOX3* participates in the activation of cytokinin biosynthesis (Azarakhsh et al., 2020). *KNOX3*, *KNOX4*, and *KNAT7* belong to the *KNOX* gene family, which may play a similar role in seed germination. Therefore, we speculate that *KNOX3* regulates seed coat mucus deposition and cuticle composition to change seed coat structure and permeability and may also regulate cytokinin biosynthesis, which probably promote the germination of *C. rigescens* seeds. Mitochondria play an important role in seed germination (Carrie et al., 2013). *GhHSP24.7* is a mitochondrial heat shock protein that modulates seed germination by regulating CytC/C1 activity and ROS generation from the mETC (Ma et al., 2019). In our study, the discovery of *mhsP70* may be key to studying the mechanism of seed germination in *C. rigescens*. Calcium signaling is critical to plant development (Midhat et al., 2018). *CML39* is a calmodulin-related protein; its mutants regulate hormone metabolism and change seed coat structure during *A. thaliana* seed germination (Midhat et al., 2018). We expect that *CML19* and *CML39* have similar functions and will provide new insights into the germination mechanism of *C. rigescens*. In conclusion, we identified four core genes that may be related to seed germination in *C. rigescens.* We plan to verify their function through transgenic methods in future studies.

For many physiological and metabolic changes during germination, starch and soluble sugar content reflect the basic level of available materials and energy supply (Lin et al., 2009). Soluble sugar and ATP produced by starch metabolism provide sufficient substrate and energy for seed germination, respectively (Styer and Cantliffe, 1984; Dirk et al., 1999). Starch and soluble sugar content and amylase activity decreased during seed germination in *C. rigescens*. Similar results were obtained in a study on *Chloris virgata* (Zhao et al., 2018). The decrease in amylase activity may be due to the decrease in starch content in the seeds, which weakened the hydrolysis activity. These results suggest that the metabolic pathways of starch and sucrose may be vital to germination. Protein products can determine the function of seed germination genes (Dong et al., 2015). Some soluble proteins are enzymes involved in various metabolic processes (Peng, 2001), and their hydrolysates participate in a series of activities during the late stage of seed germination (Onomo et al., 2010). In the present study, the decrease in soluble protein content was significant, indicating that the metabolic activity of seeds was highly active, which was conducive to their germination. We also found that the POD activity increased during this process. POD can effectively remove reactive oxygen species, reduce the activity of inert acetic acid oxidase, and accumulate IAA with increasing activity, which promoted *Styrax tonkinensis* embryo elongation (Zhao et al., 2019).

We focused on the metabolic pathways of sucrose and starch during seed germination. In our results, the metabolism of starch in *C. rigescens* seeds was possibly accelerated by upregulating the expression level of one *PYG* gene, which is consistent with the results of a study on *Cinnamomum migao* (Huang et al., 2021). The expression of three *SUS* genes in the S2–S5 period was much higher than that of other genes, indicating that *SUS* may play an important role in starch metabolism. According to previous reports, *SUS* had a similar expression during the germination of *C. henryi* (Liu et al., 2020), and the expression of *SUS4* was evidently specific to germinated seeds (Bieniawska et al., 2010). The upregulated expression of *malZ* (Tibbot et al., 1998), *INV* (Liu et al., 2020), and *HK* (Aguilera-Alvarado et al., 2019) activated sucrose metabolism. Fructokinase was an important regulator of carbohydrate metabolism (Jiang et al., 2003). Increased gene expression of the fructokinase-encoding gene promoted metabolic activity during germination (Yang et al., 2007; Han et al., 2014; Huang et al., 2021). The expression of one fructokinase gene was much higher than that of other genes in this study, and it was enhanced during S2–S5, which ensure the substrate supply of glycolysis and the tricarboxylic acid cycle. This result indicated that fructokinase may be the key enzyme in sucrose metabolism activated during seed germination. Overall, D-fructose, D-glucose, and D-glucose 6-phosphate may eventually participate in respiration and promote seed germination in *C. rigescens*. The functions of these genes need to be further studied in the future.

## Conclusion

We found that seed germination of *C. rigescens* was significantly improved under variable temperature. Physiological observations revealed that improved seed germination may be related to reduced nutrient (starch, soluble sucrose, and soluble protein) content and increased peroxidase enzyme activity. Based on the newly built PacBio full-length reference transcriptome, Illumina short read sequences were used to screen DEGs involved in the germination process. WGCNA of the DEGs identified four hub genes, *BBX24*, *KNOX3*, *mhsp70*, and *CML19*; further studies are needed to verify their roles in the seed germination of *C. rigescens*. In addition, we found that starch and sucrose metabolism pathways may be responsible for the improved germination rate under variable temperature conditions. These results lay a foundation for further research on functional genomics and genetic engineering of *C. rigescens* for improved seed germination.

## Data Availability Statement

The original contributions presented in the study are publicly available. This data can be found here: Raw sequencing data were deposited into the NCBI Short Read Archive with the accession numbers PRJNA733858 (PacBio) and PRJNA733863 (Illumina).

## Author Contributions

KT, JW, and XF conceived the study and designed the experiments. WT performed the experimental material collection. HL performed experiments and analyzed the data according to the suggestions of KT, YY, HZ, and HW. HL completed the manuscript. KT revised the manuscript. All authors read and approved the final version of the manuscript.

## Conflict of Interest

The authors declare that the research was conducted in the absence of any commercial or financial relationships that could be construed as a potential conflict of interest.

## Publisher’s Note

All claims expressed in this article are solely those of the authors and do not necessarily represent those of their affiliated organizations, or those of the publisher, the editors and the reviewers. Any product that may be evaluated in this article, or claim that may be made by its manufacturer, is not guaranteed or endorsed by the publisher.

## Footnotes

1
http://www.premierbiosoft.com

2
www.biocloud.net

3
http://cytoscape.org

## Supplementary Material

Supplementary Figure 1 Single molecular real time (SMRT) sequence length distribution. **(A)** Circular consensus (CCS) read length distribution for 1-6K size bin. **(B)** The distribution of full passes of each cDNA database. **(C)** Full-length non-chimeric (FLNC) sequences read length distribution for F01 size bin. **(D)** Consensus isoforms read length distribution for F01 size bin.
