# Supplementary figures and images for: Seed Germination Mechanism of Carex rigescens Under Variable Temperature Determinded Using Integrated Single-Molecule Long-Read and Illumina Sequence Analysis
Source: Front Plant Sci. 2022 Mar 3;13:818458. doi: 10.3389/fpls.2022.818458 (PMC8928477; doi:10.3389/fpls.2022.818458)

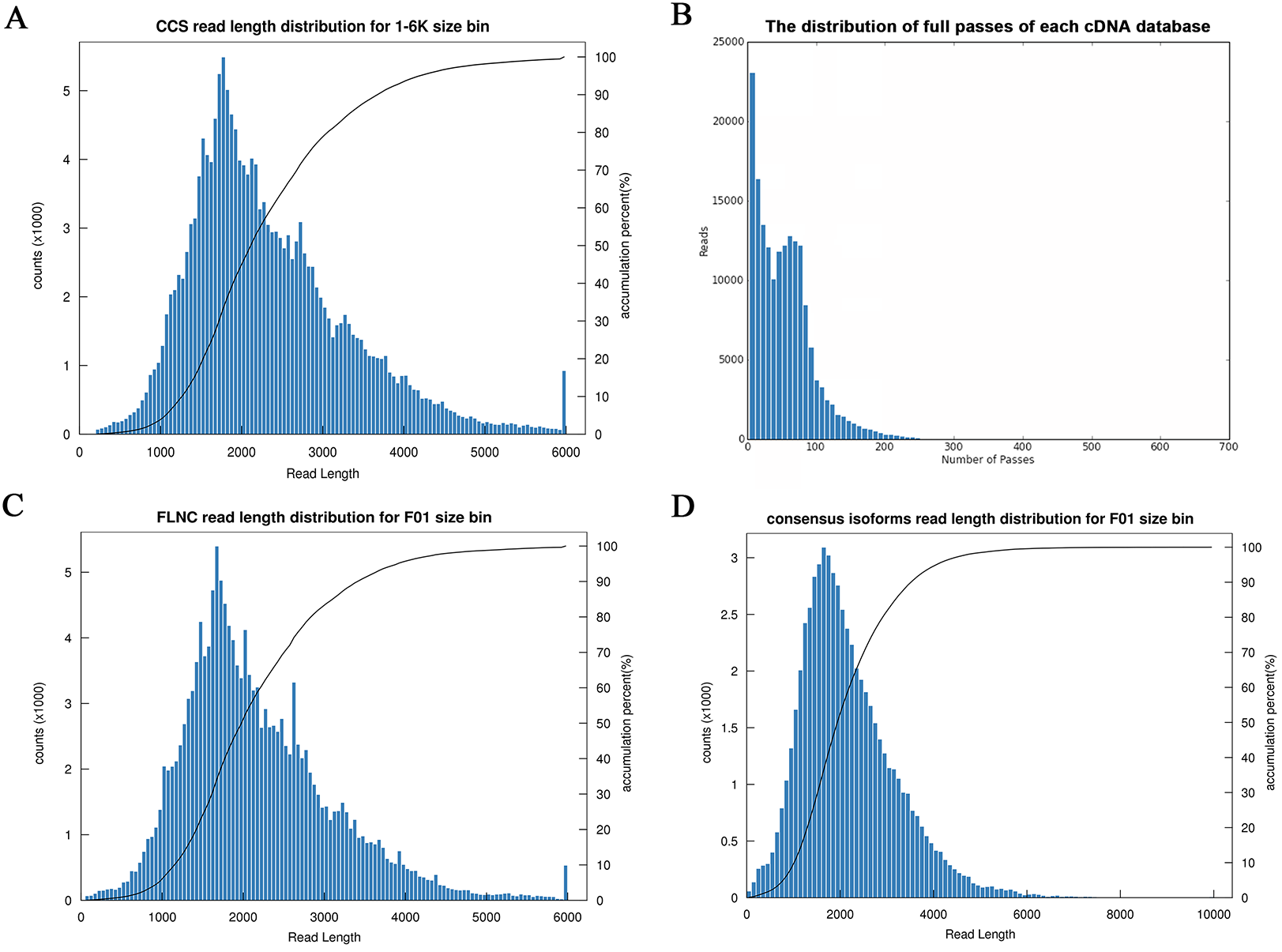

Supplement: Supplementary Figure 1 — Single molecular real time (SMRT) sequence length distribution. (A) Circular consensus (CCS) read length distribution for 1-6K size bin. (B) The distribution of full passes of each cDNA database. (C) Full-length non-chimeric (FLNC) sequences read length distribution for F01 size bin. (D) Consensus isoforms read length distribution for F01 size bin. [file Image_1.TIF]
